# ARF6 Regulates Neuron Differentiation through Glucosylceramide Synthase

**DOI:** 10.1371/journal.pone.0060118

**Published:** 2013-03-28

**Authors:** Lu Li, Marcus Ståhlman, Mikael Rutberg, Liliana Håversen, Per Fogelstrand, Linda Andersson, Malin Levin, Jan Borén

**Affiliations:** 1 Wallenberg Laboratory, Sahlgrenska University Hospital, Gothenburg, Sweden; 2 Institute of Medicine, Department of Molecular and Clinical Medicine, Sahlgrenska Academy at University of Gothenburg, Gothenburg, Sweden; Baylor College of Medicine, United States of America

## Abstract

The small GTPase ADP ribosylation factor 6 (ARF6) mediates endocytosis and has in addition been shown to regulate neuron differentiation. Here we investigated whether ARF6 promotes differentiation of Neuro-2a neuronal cells by modifying the cellular lipid composition. We showed that knockdown of ARF6 by siRNA in Neuro-2a cells increased neuronal outgrowth as expected. ARF6 knockdown also resulted in increased glucosylceramide levels and decreased sphingomyelin levels, but did not affect the levels of ceramide or phospholipids. We speculated that the ARF6 knockdown-induced increase in glucosylceramide was caused by an effect on glucosylceramide synthase and, in agreement, showed that ARF6 knockdown increased the mRNA levels and activity of glucosylceramide synthase. Finally, we showed that incubation of Neuro-2a cells with the glucosylceramide synthase inhibitor D-threo-1-phenyl-2-decanoylamino-3-morpholino-1-propanol (D-PDMP) normalized the increased neuronal outgrowth induced by ARF6 knockdown. Our results thus show that ARF6 regulates neuronal differentiation through an effect on glucosylceramide synthase and glucosylceramide levels.

## Introduction

Neuron development and differentiation are complex processes that involve dynamic cell morphology changes. It has been suggested that endosomal trafficking is crucial for actin cytoskeleton structure and neuronal cell differentiation [1]. One well-known regulator of endosomal trafficking is the small GTPase ADP ribosylation factor 6 (ARF6), which localizes to the plasma membrane and endosomal compartments [2], [3]. In addition, ARF6 has been shown to play important roles in the regulation of actin cytoskeleton and neuronal extension and branching [4], [5], [6], [7], [8], [9], [10]. However, the link between ARF6-dependent endocytosis and neuron differentiation remains unclear.

Endocytosis is also regulated by modulation of the lipid composition of cellular membranes [11], [12]. Alterations in lipid composition provide a possible mechanism for regulating endosomal cargo entry, as some proteins associate preferentially with certain types of lipids, such as sphingolipids, phospholipids and cholesterol. Interestingly, ARF6 has been shown to regulate signaling of bioactive lipids in the plasma membrane [1], [13].

In this study, we investigated whether ARF6-dependent neuron differentiation is regulated by alterations in lipid composition. We found that ARF6 knockdown resulted in increased glucosylceramide content and glucosylceramide synthase activity in Neuro-2a neuronal cells. Furthermore, we found that ARF6-dependent neuron differentiation is regulated by the altered glucosylceramide synthase activity in neuronal cells.

## Materials and Methods

### Cell culture and differentiation

Neuro-2a cells were purchased from the American Type Culture Collection (ATCC, LGC Standards, Middlesex, UK). Cells were cultured in DMEM with 10% serum and were differentiated in medium without serum.

### RT-PCR expression analyses

Total RNA was extracted with an RNeasy Kit (QIAGEN, Hilden, Germany), and cDNA was synthesized with the high-capacity cDNA Reverse Transcription Kit (Applied Biosystems, Foster City, CA). mRNA expression of genes of interest was analyzed with TaqMan real-time polymerase chain reaction in an ABI Prism 7900 HT Detection System (Applied Biosystems) and normalized to β-actin. The following TaqMan Gene Expression assays from Applied Biosystems were used: GCS (Ugcg) Mm00495925_m1, beta-Actin Mm01205647_g1, NeuroD1 Mm01946604_s1, Rbfox3 Mm01248771_m1 and 36B4 Mm007725448_s1.

### Transfection of siRNA and plasmids

Neuro-2a cells were cultured at 30% confluence and transfected with 100 pmol/l target-specific or scrambled control siRNA (50 nmol/l) using Lipofectamine RNAiMAX (Invitrogen, Carlsbad, CA) according to the manufacturer's protocol. The siRNAs used were from Applied Biosystems (siARF6-1: sense AUCUGACAUUUGACACGAATT, antisense UUCGUGUCAAUGUGAGAUCA; siARF6-2: sense GCAAGACAACGAUCCUGUATT, antisense UACAGGAUCGUUGUCUUGCCG).

Plasmids for ARF6-cyan fluorescent protein (CFP), T27N ARF6-CFP and Q67L ARF6-CFP were purchased from Addgene (Cambridge, MA). Neuro-2a cells were transfected with plasmids at 70–80% confluence using jetPRIME (Polyplus-transfection, Illkirch, France) according to the manufacturer's instruction. Transfection efficiency (determined by CFP by using fluorescent microscopy) was around 50%.

### Quantification of outgrowth of Neuro-2a cells

The neuronal outgrowth was assessed in Neuro-2a cells that were cultured in DMEM with 10% serum. Long outgrowths from Neuro-2a cells were defined as having a length at least double that of the cell diameter. Randomly selected micrographs of 100–300 cells from 4 different experiments were analyzed for percentage of cells with outgrowth or long outgrowth.

### Lipid analysis

Lipids were extracted from Neuro-2a cells using the Folch procedure [14]. Heptadecanoyl (C17:0)-containing internal standards of phosphatidylcholine, sphingomyelin, and ceramide and dodecanoyl (C12:0)-containing glucosylceramide were added during the extraction. The extract was evaporated using nitrogen, reconstituted in chloroform∶methanol (2∶1) and stored at −20°C until further analysis.

Phosphatidylcholine, phosphatidylethanolamine, phosphatidylserine and sphingomyelin were quantified as described [15] using direct infusion on a QTRAP 5500® mass spectrometer (ABSciex, Toronto, Canada) equipped with the chip-based nanoESI source TriVersa NanoMate (Advion Biosciences, Ithaca, NJ). Mass spectrometry data files were processed using Lipid Profiler™ [16]. The lipids were quantified using their respective internal standard and normalized against the cellular protein content.

Ceramide and glucosylceramide were analyzed using straight-phase HPLC coupled to a Quattro Premier XE triple quadrupole mass spectrometer (Waters, Milford, MA). The lipids were separated using a Sunfire 150×2.1 silica column with 3 µm particles (Waters). The mobile phase A was isohexane∶isopropanol (95∶5) and the mobile phase B was isohexane∶isopropanol:50 mmol/l ammonium formate in water (25∶65∶10). The gradient went from 100% A (held for 1 min) to 100% B in 5 min. After 4 min at 100% B, the gradient returned to 100% A and the column was equilibrated for 3 min. Thus, the total runtime was 13 min. The flow rate was 500 µl/min. A postcolumn flow of methanol∶isopropanol (1∶1) at 100 µl/min was used to ensure optimal ionization of the lipids in the ion source. Samples were injected in mobile phase A. Ceramide and glucosylceramide were detected using multiple reaction monitoring, quantified using external standards and normalized against their respective internal standard and the cellular protein content.

### Glucosylceramide synthase activity

The activity of glucosylceramide synthase was measured by following the conversion of fluorescently labeled ceramide 6-*N*-[7-nitrobenz-2-oxa-1,3-diazol-4-yl] aminocaproyl-sphingosine (NBD C6-ceramide) to NBD C6-glucosylceramide in cells, as described [17], [18]. In brief, cells were washed three times with Hank's balanced salt solution (GIBCO, Invitrogen, Carlsbad, CA) and then incubated with 5 µmol/l NBD C6-ceramides with 5 µmol/l BSA at 4°C for 3 h. The cells were harvested and the lipids were extracted using chloroform∶methanol 2∶1. The lipids were separated by thin layer chromatography (TLC) (chloroform∶methanol∶ammonium 65∶35∶5) and the fluorescent NBD-glucosylceramide was measured and quantified using fluorescent image acquisition system Fusion FX7 (Peqlab, Sarisbury Green, UK).

### Statistics

Data are presented as mean ± s.e.m. Statistical significance was determined with Student's *t* test or one-way ANOVA with Dunnett's post-hoc test.

## Results and Discussion

### ARF6 knockdown stimulates differentiation of Neuro-2a cells

It has previously been observed that inactivation of ARF6 by overexpression of a GAP or a dominant-negative ARF6 promotes neuron differentiation, as shown by increased neuronal outgrowth, in various neuronal cell systems [9], [19]. Furthermore, activation of ARF6 by expression of the dominant-active ARF6-Q67L decreases the neuronal outgrowth [6], [8], [10], [19].

**Figure 1:**
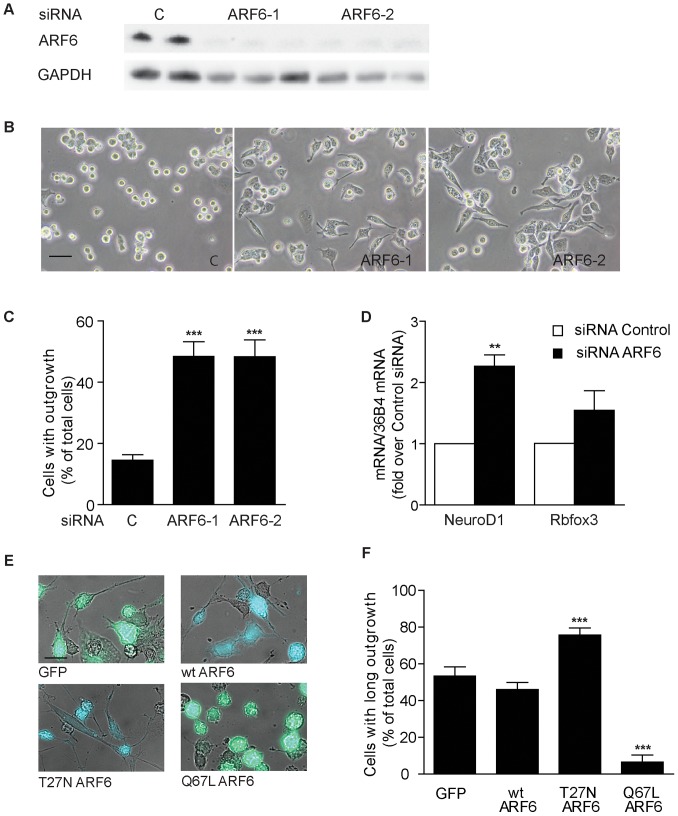
ARF6 is important for the differentiation of Neuro-2a cells. (**A–D**) Neuro-2a cells were transfected with control (c) or ARF6 siRNA in medium with 10% FCS and analyzed after 48 h. (**A**) ARF6 protein levels in cell lysate after siRNA treatment. (**B**) Representative micrographs showing neuronal outgrowth from Neuro-2a cells after knockdown of ARF6 using siRNA. Bar size, 40 µm. (**C**) Quantification of neuronal outgrowth from Neuro-2a cells after knockdown of ARF6 using siRNA. (**D**) mRNA expression of differentiation markers NeuroD1 and Rbfox3 in RNA extracted from Neuro-2a cells (*n* = 2 for control and *n* = 6, 3 combined siRNAs, **P<0.01 *vs* control) (**E**) Representative micrographs showing long outgrowth from Neuro-2a cells transfected with green fluorescent protein (GFP), wildtype ARF6, dominant-negative T27N ARF6 or dominant-active Q67L ARF6 and analyzed after 48 h. Bar size, 20 µm. (**F**) Quantification of long outgrowth from Neuro-2a cells transfected with green fluorescent protein (GFP), wildtype ARF6, dominant-negative T27N ARF6 or dominant-active Q67L ARF6 and analyzed after 48 h. *n* = 4 per group, ***P<0.001 *vs* control/GFP.

In this study, we used Neuro-2a neuronal cells and knocked down ARF6 with siRNA to study the effects on neuron differentiation (Figure 1). We found that 48 hours after transfection with ARF6 siRNA, ARF6 protein was almost totally abolished (Figure 1A) and ARF6-deficient cells displayed significantly increased neuronal outgrowth (Figure 1B–C). In addition, the mRNA expression of the differentiation marker NeuroD1, which has been shown to be increased following neuronal differentiation [20], was upregulated (Figure 1D). In agreement with previous reports, overexpression of mutant constructs in Neuro-2a cells confirmed that inactivation of ARF6 (T27N-ARF6) increased neuronal outgrowth and activation of ARF6 (Q67L-ARF6) decreased neuronal outgrowth in our model system (Figure 1E–F). Our results show that ARF6 inactivation using siRNA in Neuro-2a cells is a valid cellular system to investigate ARF6-dependent neuron differentiation and that it is as effective as using dominant negative constructs. Thus, we can avoid using plasmid constructs for the lipidomics analysis since we have previously experienced that overexpression using DNA plasmids may cause an inflammatory response and thereby affect the lipidome (data not shown).

### ARF6 knockdown increases glucosylceramide levels and decreases sphingomyelin levels in Neuro-2a cells

**Figure 2:**
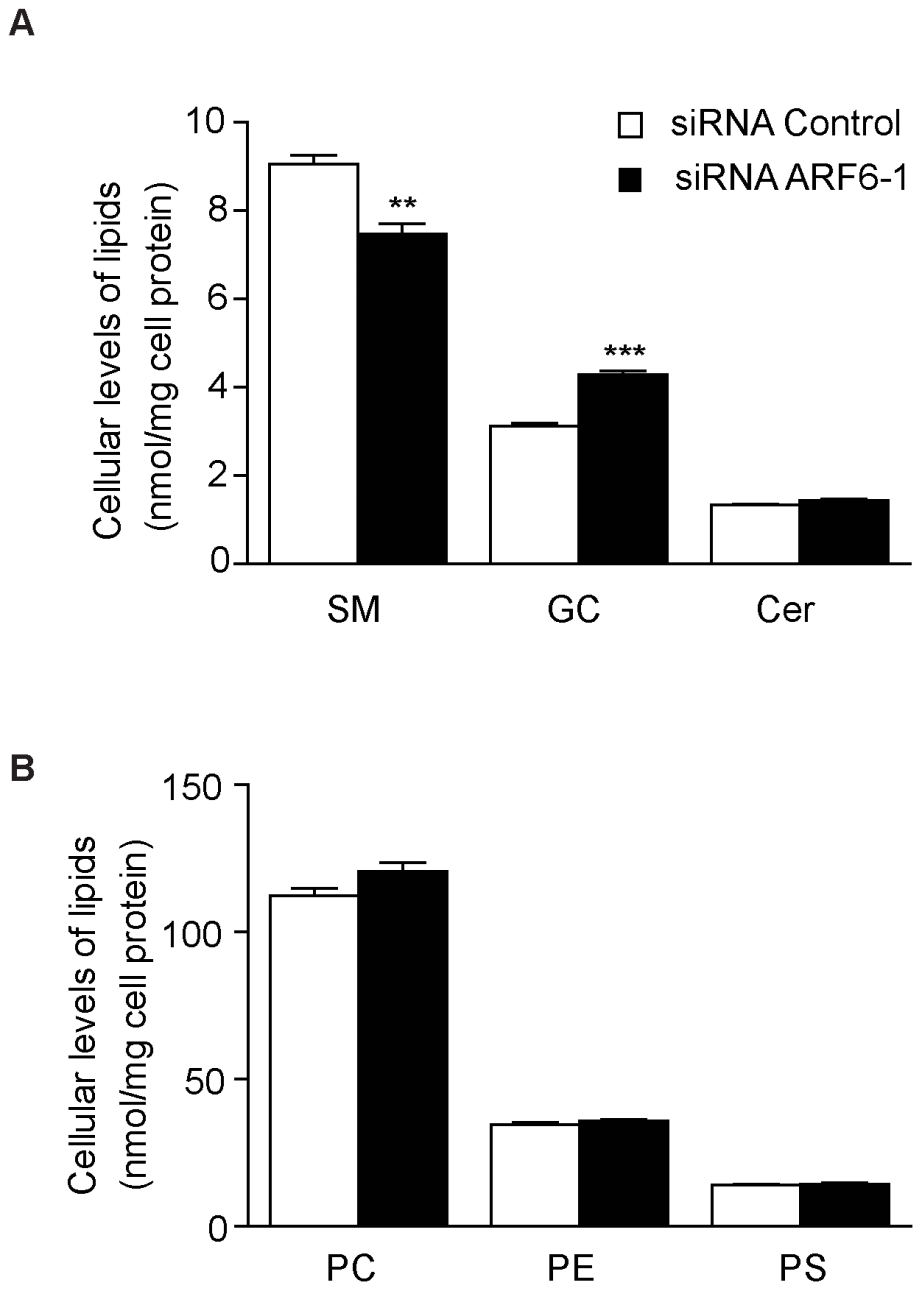
ARF6 knockdown increases glucosylceramide levels and decreases sphingomyelin levels in Neuro-2a cells. Neuro-2a cells were transfected with control (c) or ARF6 siRNA in medium with 10% FCS and lipids were extracted after 48 h. (**A**) Cellular levels of ceramides (Cer), sphingomyelin (SM) and glucosylceramide (GC) and (**B**) phosphatidylcholine (PC), phosphatidylethanolamine (PE) and phosphatidylserine (PS) were analyzed as described in the methods section. *n* = 4 per group, **P<0.01, ***P<0.001 *vs* control.

To investigate whether ARF6 knockdown affects the lipid composition of Neuro-2a cells, we performed a lipidomics analysis of Neuro-2a cells transfected with control or ARF6 siRNA. Interestingly, we showed that ARF6 knockdown resulted in significantly increased levels of glucosylceramide and significantly decreased levels of sphingomyelin but did not change ceramide levels (Figure 2A). Levels of phosphatidylcholine, phosphatidylethanolamine and phosphatidylserine were unaltered by ARF6 knockdown (Figure 2B).

Our results indicate that ARF6 is a regulator of the sphingolipid composition of Neuro-2A cells. Numerous studies have shown that glucosphingolipids, especially gangliosides, are important for neuron differentiation and development. The ganglioside GM1, for example, has been shown to promote neuron outgrowth in both primary neurons and neuronal cell lines [21], [22], [23], [24]. In addition, syndromes in humans with disturbed glucosphingolipid expression and metabolism, such as lysosomal storage disease, are linked to brain dysfunction [24], [25].

### ARF6 knockdown increases glucosylceramide synthase mRNA and activity

**Figure 3:**
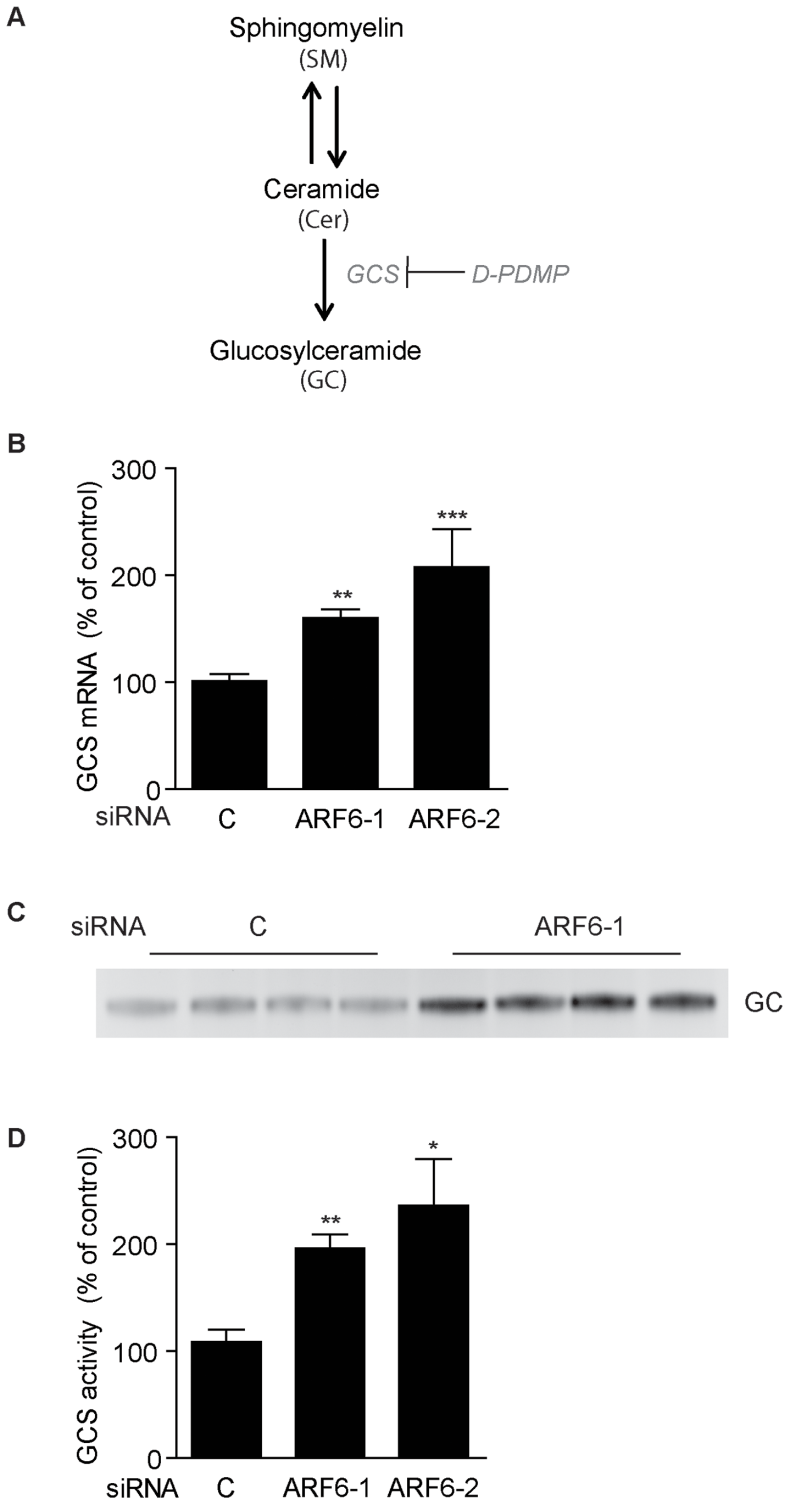
ARF6 knockdown increases cellular glucosylceramide levels through increased glucosylceramide synthase activity in Neuro-2a cells. (**A**) Schematic overview of the sphingolipid synthesis pathway. (**B–D**) Neuro-2a cells were transfected with control (c) or ARF6 siRNA in medium with 10% FCS and analyzed after 48 h. (**B**) mRNA expression of glucosylceramide synthase (GCS) in RNA extracted from Neuro-2a cells (*n* = 6 per group). (**C**) Representative image of a TLC plate from a GCS activity assay showing increased levels of synthesized NBD-glucosylceramide in Neuro-2a cells transfected with ARF6 siRNA. (**D**) Quantification of increased glucosylceramide synthase activity after ARF6 knockdown (*n* = 4 per group). *P<0.05, **P<0.01, ***P<0.001 *vs* control.

Glucosylceramide and sphingomyelin are synthesized from ceramide in the Golgi apparatus (Figure 3A) [26], [27]. Because ceramide levels were unchanged by ARF6 knockdown, we speculated that the shift in the relative levels of sphingomyelin and glucosylceramide could be caused by an effect of ARF6 knockdown on glucosylceramide synthase. In agreement with our hypothesis, we observed significantly increased mRNA expression of glucosylceramide synthase in Neuro-2a cells transfected with ARF6 siRNA (Figure 3B). In addition, we tested whether glucosylceramide synthase activity was affected by ARF6 knockdown by following the synthesis of glucosylceramide from fluorescently labeled NBD-C6-ceramide. Importantly, we found that glucosylceramide synthase activity was increased by 80–100% after ARF6 knockdown (Figure 3C–D).

Increased glucosylceramide synthase activity will promote the conversion of ceramide to glucosylceramide. Our results thus indicate that glucosylceramide synthase activity is important for the ARF6-regulated modification of sphingolipid levels in Neuro-2a cells.

### ARF6 regulates neuron differentiation through glucosylceramide synthase activity

**Figure 4:**
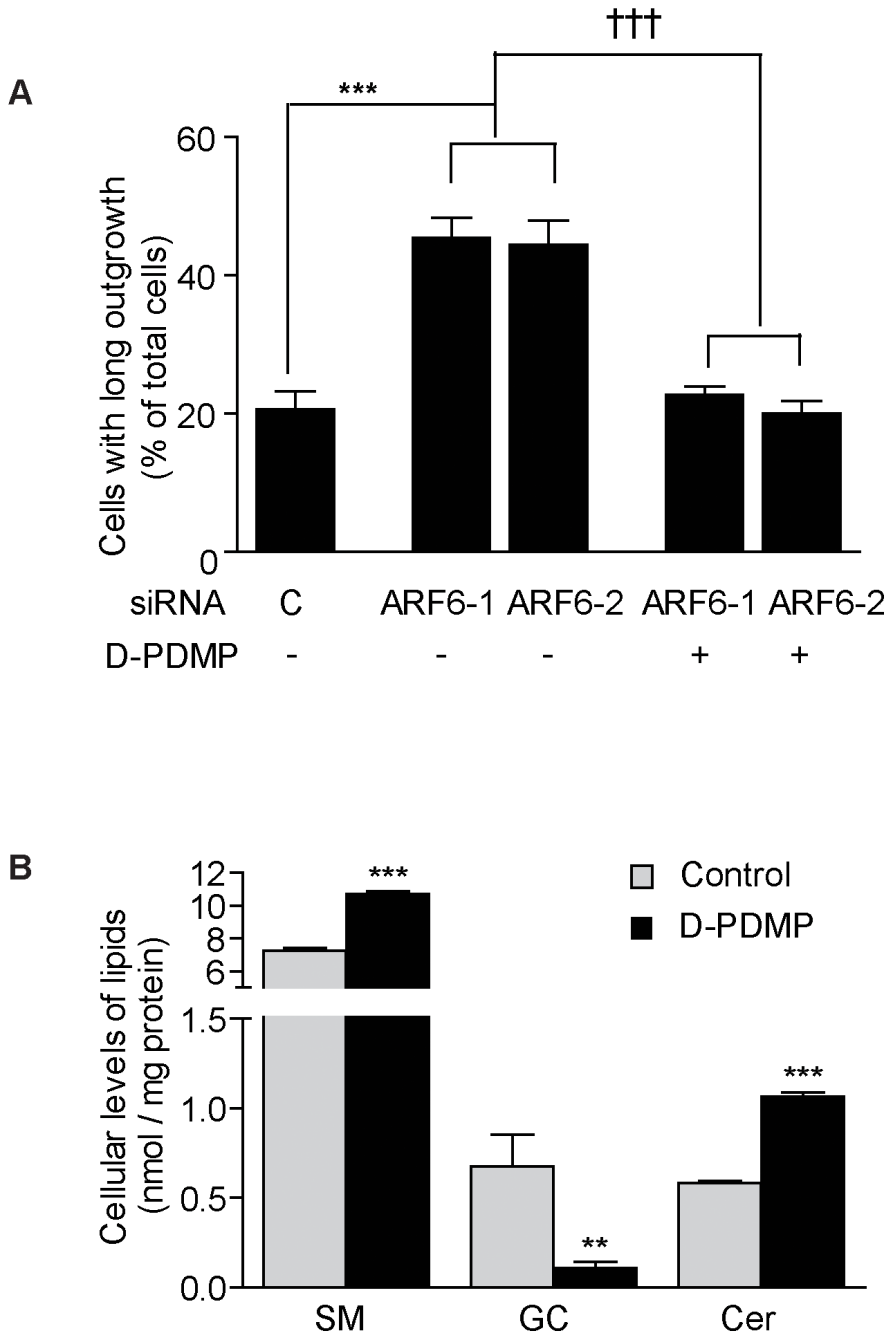
ARF6-dependent neuronal differentiation is normalized after inhibition of glucosylceramide synthase. (**A**) Quantification of long outgrowth from Neuro-2a cells transfected with control or ARF6 siRNA in medium with 10% FCS for 48 h and then differentiated for 24 h in medium without serum in the absence or presence of D-PDMP (10 µmol/l). *n* = 4 per group, ***P<0.001 *vs* control; †††*P*<0.001 *vs* absence of D-PDMP. (**B**) Cellular levels of ceramides (Cer), sphingomyelin (SM) and glucosylceramide (GC). *n* = 4–5 per group, **P<0.01 *vs* control, ***P<0.001 *vs* control.

Finally, we tested if ARF6 regulates differentiation of Neuro-2a cells through an effect on glucosylceramide synthase. We knocked down ARF6 for 48 h to stimulate neuronal outgrowth and then incubated the cells in the presence or absence of the glucosylceramide synthase inhibitor D-threo-1-phenyl-2-decanoylamino-3-morpholino-1-propanol (D-PDMP) for 20 h. As expected, the number of cells with long outgrowths increased significantly following ARF6 knockdown (Figure 4A). Interestingly, this increased neuronal outgrowth was normalized after incubation with D-PDMP (Figure 4A). Lipid analysis revealed that D-PDMP almost totally abolished glucosylceramide accumulation in Neuro-2a cells (Figure 4B) as expected. In contrast, ceramide and sphingomyelin levels increased after D-PDMP treatment (Figure 4B). Our results clearly indicate that ARF6 regulates neuronal differentiation through effects on glucosylceramide synthase activity.

The lipid composition of neuronal membranes has been shown to be important for actin structure and endosomal trafficking [1]. Indeed, glucosylceramide synthase activity has been suggested to be involved in the differentiation of neuronal cells and D-PDMP has previously been shown to inhibit the outgrowth of Neuro-2a cells and rat pheochromocytoma PC12 cells [21], [28], [29]. D-PDMP may affect neuronal differentiation by depleting the levels of glucosylceramides or their products gangliosides in neuronal membranes.

In conclusion, our results indicate that ARF6 regulates neuronal differentiation through modulation of glucosylceramide levels.
